# PD42 Systematic Review Of The Comprehensive Approach To Gender Reassignment Surgery

**DOI:** 10.1017/S026646232510175X

**Published:** 2025-12-29

**Authors:** Santiago Torales, Darío García, Emanuel Medina

## Abstract

**Introduction:**

Access to gender reassignment surgery (GRS) as an isolated process would not imply achieving clinical and satisfaction results in transgender people. Public subsystems face a shortage of trained professionals, a lack of protocols, fragmented care, and economic and geographic barriers. This review sought evidence on integrated and equitable care models for access to GRS.

**Methods:**

A systematic review (SR) was conducted on the effectiveness and safety of GRS prevalent in Argentina (vulvovaginoplasty [VVP] and mastectomy [MRP]) in transgender people older than 16 years. The SR also considered final perceptions from an integrated and comprehensive management approach. The Web of Science and MEDLINE databases were consulted, prioritizing clinical trials, SRs, health technology assessments, and relevant individual studies from 1988 to 2024, using a rapid umbrella review methodology. The results of the evidence were ordered by relevant outcomes (aesthetic, satisfaction, complications, mental health) within the framework of integrated models applicable to inclusive and equitable health policies.

**Results:**

For MRP, six SRs (two specific, four general) and six cohort studies (three specific, three general) were included. For VVP, 16 SRs (nine specific, seven general) and 38 cohort studies (35 specific, three general) were included. Techniques for each surgery were described, citing results about complications (71%), quality of life (48%), mental health (16%), aesthetic aspects (58%), and satisfaction (73%), but only 0.2 percent considered them within frameworks of integrated models. With a lack of quality evidence, challenges persist in unifying classifications of outcomes over time and generalizing findings to different health systems.

**Conclusions:**

The recommendations of scientific societies and current legal frameworks suggest access to GRS as an advanced step within comprehensive care processes for transgender people. The evidence showed a divergence between this approach and the findings, with data on complications and satisfaction predominating, less information on associated quality of life and mental health, and few references to integrated models.

